# OsSND2, a NAC family transcription factor, is involved in secondary cell wall biosynthesis through regulating MYBs expression in rice

**DOI:** 10.1186/s12284-018-0228-z

**Published:** 2018-05-31

**Authors:** Yafeng Ye, Kun Wu, Jianfeng Chen, Qian Liu, Yuejin Wu, Binmei Liu, Xiangdong Fu

**Affiliations:** 1 Institute of Technical Biology and Agricultural Engineering, Hefei Institutes of Physical Science, Chinese Academy of Sciences, Hefei, Anhui 230031 People’s Republic of China; 2 Key Laboratory of High Magnetic Field and Ion Beam Physical Biology, Hefei Institutes of Physical Science, Chinese Academy of Sciences, Hefei, Anhui 230031 People’s Republic of China; 3 State Key Laboratory of Plant Cell and Chromosome Engineering, Institute of Genetics and Developmental Biology, Chinese Academy of Sciences, Beijing, 100101 China

**Keywords:** Secondary cell wall (SCW), Rice, Cellulose synthesis, Transcription factor (TF), NAC, MYB

## Abstract

**Background:**

As one of the most important staple food crops, rice produces huge agronomic biomass residues that contain lots of secondary cell walls (SCWs) comprising cellulose, hemicelluloses and lignin. The transcriptional regulation mechanism underlying SCWs biosynthesis remains elusive.

**Results:**

In this study, we isolated a NAC family transcription factor (TF), OsSND2 through yeast one-hybrid screening using the secondary wall NAC-binding element (SNBE) on the promoter region of *OsMYB61* which is known transcription factor for regulation of SCWs biosynthesis as bait. We used an electrophoretic mobility shift assay (EMSA) and chromatin immunoprecipitation analysis (ChIP) to further confirm that OsSND2 can directly bind to the promoter of *OsMYB61* both in vitro and in vivo. OsSND2, a close homolog of AtSND2, is localized in the nucleus and has transcriptional activation activity. Expression pattern analysis indicated that *OsSND2* was mainly expressed in internodes and panicles. Overexpression of OsSND2 resulted in rolled leaf, increased cellulose content and up-regulated expression of SCWs related genes. The knockout of *OsSND2* using CRISPR/Cas9 system decreased cellulose content and down-regulated the expression of SCWs related genes. Furthermore, OsSND2 can also directly bind to the promoters of other MYB family TFs by transactivation analysis in yeast cells and rice protoplasts. Altogether, our findings suggest that OsSND2 may function as a master regulator to mediate SCWs biosynthesis.

**Conclusion:**

*OsSND2* was identified as a positive regulator of cellulose biosynthesis in rice. An increase in the expression level of this gene can improve the SCWs cellulose content. Therefore, the study of the function of OsSND2 can provide a strategy for manipulating plant biomass production.

**Electronic supplementary material:**

The online version of this article (10.1186/s12284-018-0228-z) contains supplementary material, which is available to authorized users.

## Background

Plant cell wall is a unique structure that plays an important role in plant growth and development. The cell wall provides mechanical strength to the plant body and responses to environmental stimuli, such as pathogen invasion (Underwood, 2012) and stress response (Tenhaken, 2014). Plants exhibit two typical types of cell walls, namely, the primary cell walls (PCWs) that surround all cells and the secondary cell walls (SCWs), a thickened structures observed in specific cell types, such as xylem vessels and fibers (Keegstra, 2010). SCWs not only provide mechanical strength to these cells, but also greatly contribute to the bulk of renewable plant biomass (Burton and Fincher, 2014). SCWs mainly composes cellulose, hemicelluloses and lignin. Cellulose is composed of unbranched β-1, 4-glucans, and cellulose microfibrils form the main load-bearing network (Somerville, 2006). Hemicelluloses belong to a group of heterogeneous polysaccharides such as xylan, glucan, mannan, and mixed-linkage glucan (Pauly et al., 2013). Lignin is a complex phenylpropanoid polymer that provides mechanical strength to specific cell types (Boerjan et al., 2003). The understanding of the mechanism underlying SCWs biosynthesis may provide a strategy for manipulating plant biomass production.

In the past decades, many genes involved in SCWs biosynthesis have been cloned and characterized in both dicot and monocot plants. Cellulose is synthesized in the plasma membrane by the cellulose synthase complex (CSC), which contains at least three different cellulose synthases, encoded by *CESA* genes (Somerville, 2006). In *Arabidopsis*, *CESA4*, *CESA7*, and *CESA8* genes are essential for SCWs cellulose biosynthesis (Taylor et al., 2003; Taylor et al., 2000; Taylor et al., 1999). Close homologs of CESA4, CESA7 and CESA8 are required for SCWs cellulose biosynthesis in rice, and mutations in any of these genes may cause a dramatic decrease in the SCWs cellulose content, resulting in the brittle culm phenotype (Song et al., 2013; Tanaka, 2003; Zhang et al., 2009). In addition to these *CESA* genes, some other genes are also involved in SCWs cellulose biosynthesis and assembly, such as the *Arabidopsis KORRIGAN* (*KOR*) gene, that encodes for an endo-β-1, 4-glucanase. Mutations in this gene causes reduction in the cellulose content of both PCWs and SCWs (Szyjanowicz et al., 2004). In rice, several *Brittle Culm* (*BC*) genes are involved in SCWs cellulose biosynthesis and mutations of these genes are shown to reduce the cellulose content and mechanical strength, leading to the brittle culm phenotype (Kotake et al., 2011; Wu et al., 2012; Zhang et al., 2010; Zhou et al., 2009). Xylan and mannan are the major hemicelluloses in SCWs, and they are synthesized in the Golgi apparatus and transported to the plasma membrane via Golgi vesicles (Pauly et al., 2013). In *Arabidopsis*, glycosyltransferase families have been implicated in hemicelluloses biosynthesis. Lignin is a complex polymer made up of *p*-hydroxylphenyl (H), guaiacyl (G), and syringyl (S) units of lignin (Kumar et al., 2016). The monolignols are synthesized through the phenylpropanoid pathway within cells and then transported into cell walls, where they are polymerized into lignin via oxidative reactions catalyzed by oxidases, such as laccases and peroxidases (Boerjan et al., 2003). Several genes involved in SCWs biosynthesis have been reported, however the spatiotemporal expression of these genes remains unclear.

In *Arabidopsis*, a detailed transcriptional regulation mechanism of SCWs biosynthesis has been reported. A transcriptional network comprising two large family transcription factors (TFs), NAC and MYB, are involved in SCWs biosynthesis (Zhong and Ye, 2015). In this transcriptional network, a group of NAC family TFs, including NAC SECONDARY WALL THICKENING PROMOTING FACTOR1 (NST1), NST2, NST3 (also called as SND1, SECONDARY WALL-ASSOCIATED NAC DOMAIN PROTEIN1), VASCULAR-RELATED NAC-DOMAIN6 (VND6), and VND7, function as the top-level master switches of SCWs biosynthesis in fibers and/or vessels (Zhong and Ye, 2015). These factors directly regulate the expression of a battery of downstream TFs, including *SND2*, *SND3*, *MYB20*, *MYB42*, *MYB46*, *MYB52*, *MYB54*, *MYB58*, *MYB63*, *MYB83*, *MYB85* and *MYB103*. Of these, MYB46 and its close homolog MYB83 act as the secondary-layer master switches to regulate SCWs biosynthesis (Hussey et al., 2013). MYB46 and MYB83 also regulate the expression of the direct targets of SND1 and its homologs, NST1, NST2, VND6 and VND7 (McCarthy et al., 2009; Zhong et al., 2007). All of these NAC and MYB TFs collectively regulate the biosynthetic genes for cellulose, xylan and lignin. The SCWs NAC family TFs activate the downstream targets through binding to a 19 base pair (bp) sequence, known as SCWs NAC-binding element (SNBE) (McCarthy et al., 2014; Zhong et al., 2010). MYB46 and MYB83 bind to a 7 bp consensus sequence, termed as SCWs MYB-responsive element (SMRE) to regulate the expression of target genes (Zhong and Ye, 2012).

Rice is one of the most important staple food crops and produces a large amount of agronomic biomass residues, which may be a potential source of bio-energy. Nevertheless, a few TFs involved in SCWs biosynthesis have been reported in rice. *OsCEF1*, which encodes the OsMYB103L, regulates SCWs biosynthesis by directly binding to the promoter of *CESAs* and *BC1* genes (Ye et al., 2015). The *cef1* mutant shows reduction of cellulose content, and the culm is fragile (Ye et al., 2015). OsMYB61 directly binds to the *CESA* promoters and regulates their expression, and OsMYB61 can be activated by the SCWs NAC families, including NAC29 and NAC31 (Huang et al., 2015). Therefore, to unveil the master transcriptional mechanism of SCWs biosynthesis in rice may provide valuable approach for genetically modifying grass crops for biofuel production.

In this study, we isolated a NAC family TF, named OsSND2 using yeast one-hybrid screening with the SNBE site in the promoter region of *OsMYB61* as bait. We demonstrated that OsSND2 directly binds to the promoter of *OsMYB61* in vitro and in vivo, and regulates its expression. Furthermore, molecular characterization of *OsSND2* suggested that it functions as a master regulator to directly mediate the expression of other MYBs and facilitate cellulose biosynthesis.

## Methods

### Plant materials and growth conditions

The all rice (*Oryza sativa*) plants were used in this study, including the *japonica* cultivar wild-type plants, wuyunjing7 (WYJ7) and the overexpression and knockdown of OsSND2 transgenic plants were grown in the experimental fields at the Institute of Technical Biology and Agriculture Engineering, Hefei Institute of Physical Science, Chinese Academy of Sciences (Hefei, China) and Sanya (Hainan province, China) during the natural growing season.

### Yeast one-hybrid screening

Five *OsMYB61* bait fragments of pMYB61–1 (− 1946, − 1258), pMYB61–2 (− 1607, − 1258), pMYB61–3 (− 1258, − 897), pMYB61–4 (− 870, − 356) and pMYB61–5 (− 356, − 1) were cloned into the pHIS2 vector between EcoRI and SacI sites and integrated into the genome of yeast strain Y187 (*MATα*, *ura3–52*, *his3–200*, *ade2–101*, *trp1–901*, *leu2–3*, *112*, *gal4∆*, *met*^*−*^, *gal80∆*, *URA3*:: *GAL1*_*UAS*_*-GAL1*_*TATA*_*-lacZ*, *MEL1*). For the self-activation test, promoter bait strains were grown on the SD/−Trp, -His (a synthetic Trp and His dropout medium) media in the presence of 0 mM, 10 mM, 30 mM and 50 mM 3-aminotriazole (3-AT). We performed the yeast one-hybrid screening using the BD Matchmaker One-hybrid Library Construction and Screening Kit (K1617–1, Clontech) according to the user manual (PT3529–1, Clontech). The cDNA library of the internodes tissue was constructed with the pGADT7-Rec2 vector (Clontech). The promoter bait strains were then mated with the “pGADT7-Rec2-cDNA” library and screened on the SD/−Leu -Trp -His selection media containing 30 mM 3-AT. Positive colonies were selected for yeast plasmid isolation or PCR with primers AD-F and AD-R. The PCR was performed according to the following program, 95 °C 5 min, 95 °C 30 s, 56 °C 30 s, 72 °C 2 min, 36 cycles, 72 °C 10 min, 12 °C pause.

### Bioinformatics analysis of *OsSND2*

A search for OsSND2 homologs in rice and Arabidopsis was performed using the NCBI BLAST server (http://blast.ncbi.nlm.nih.gov/Blast.cgi). The alignment was performed using DNAMAN software. An unrooted phylogenetic tree of OsSND2 homologs in rice and Arabidopsis was constructed using MEGA5 software with 1000 bootstrap replications (Tamura et al., 2011). The co-expression analysis of OsSND2 with candidates in cell wall synthesis was performed using the expressing database at http://www.ricearray.org.

### Subcellular localization of OsSND2

To observe the subcellular localization of OsSND2, a green fluorescent protein (GFP) fused to the C-terminus of OsSND2 and inserted into the *pCAMBIA1300* between the KpnΙ and BamHΙ sites to create the *35S::OsSND2-GFP* vector, which was transformed into rice protoplasts by polyethylene glycol (PEG) mediated transformation method. The subcellular distribution of the OsSND2-GFP protein was observed using confocal laser scanning microscope (Leica TCS SP5).

### Binary vectors construction and rice transformation

For the overexpression construct of *OsSND2*, the full-length coding sequence of OsSND2 was amplified using gene-specific primers, OE-F, 5’-CCAAGCTTATGACGTGGTGCAACAGCTT-3′ and OE-R, 5’-CGGGATCCTCAAGGGCCACCAAAGCTGT-3′, which contain HindΙΙΙ and BamHΙ restriction sites. The PCR fragment was cloned into the intermediate vector N-Tagged SK (−), which encodes Myc-tag protein. Then, the sequencing-confirmed vector was digested using KpnΙ/BamHΙ and inserted into the *pCAMBIA2300* between the KpnΙ and BamHΙ sites to create the *p35S::Myc-OsSND2* vector.

We used CRISPR/Cas9 system for creating *snd2* mutants. The CRISPR/Cas9 binary vectors were constructed as previously described (Ma et al., 2015). The Cas9 plant expression vector (*pYLCRISPR/Cas9Pubi-H*) and sgRNA expression vector (*pYLgRNA*) were provided by Prof. Yao-Guang Liu (South China Agricultural University). We selected the Target1 (CAGCGACGTCCGCACCGCCG) and Target2 (GGAGGGGCACATCTTGACG) in the first exon of *OsSND2* (Fig. 5a) as candidate target sequences according to the design principles of the target sequences in the CRISPR/Cas9 system. Then, they were ligated into two sgRNA expression cassettes of a Cas9 binary vector, driven by OsU6 and OsU3 promoters, respectively.

These constructs were introduced into a *japonica* cultivar, wuyunjing7 (WYJ7) by the *Agrobacterium*-mediated transformation procedure as described previously (Raineri et al., 1990).

### RNA extraction and quantitative real-time PCR (qRT-PCR)

Total RNA was extracted from various rice tissues using TRIzol reagent (Invitrogen), as described previously (Wadsworth et al., 1988). The first strand of cDNA was synthesized using a reverse transcriptional kit (TransGen). qRT-PCR was performed using relevant primers and qRT-PCR kit (TransGen) on a quantitative 7500 PCR system (ABI). All assays were repeated at least three times, the *Actin1* gene was used as an internal control.

### Electrophoretic mobility shift assay (EMSA)

The coding sequence of *OsSND2* was amplified and cloned into the *pGEX-4 T-1* vector (GE Healthcare). GST and GST-OsSND2 fusion proteins were purified as described previously (Wang et al., 2015). DNA fragments for EMSA were obtained by PCR amplification and labeled using a biotin labeling kit (Invitrogen). DNA gel shift assays were performed using the LightShift Chemiluminescent EMSA kit (Thermo Fisher Scientific).

### Chromatin immunoprecipitation (ChIP) analysis

The above-ground portion of *p35S::Myc-OsSND2* transgenic rice plants was harvested between 2 and 3 g after growth on soil for 3 to 4 weeks and immediately cross-linked with 1% formaldehyde under vacuum for 15 min at 15–25 °C. The cross-linking was stopped by adding glycine to the final concentration of 0.125 M for 5 min under vacuum. The cross-linked samples were rinsed twice with double distilled water. The further ChIP assay based on an antibody to Myc (9E10, Santa Cruz Biotechnology) was performed as described previously (Wang et al., 2015). Chromatin samples without Myc antibody immunoprecipitation were used as the control. Enrichment of DNA fragments was determined using qRT-PCR analysis performed on three biological replicates. The *Actin1* gene exon used as negative controls.

### Transactivation analysis in yeast cells and Rice protoplasts

Transactivation analysis in yeast was performed as described previously (Wang et al., 2012). The full length coding sequence of *OsSND2* was amplified and cloned into *pGBKT7* vector, and then transformed into the yeast strain AH109 (*MATa*, *trp1–901*, *leu2–3*, *112*, *ura3–52*, *his3–200*, *gal4∆*, *gal80∆*, *LYS2::GAL1*_*UAS*_*-GAL1*_*TATA*_*-HIS3*, *GAL2*_*UAS*_*-GAL2*_*TATA*_*-ADE2*, *URA3::MEL1*_*UAS*_*-MEL1*_*TATA*_*-lacZ*, *MEL1*). The empty *pGBKT7* (BD) and fusing the GAL4 vectors were used as negative and positive controls, respectively. The transactivation activity was evaluated according to the growth on SD/−Trp and SD/−Trp –His -Ade.

Transactivation analysis was also performed in rice protoplasts as described previously (Wang et al., 2015). For the effecter vector, the full length coding sequence of *TF*s were amplified and fused with GAL4 binding domain (GAL4BD). The empty GAL4BD and fused with VP16 were used as negative and positive controls, respectively. For the reporter vectors, the *pUC19* containing the firefly luciferase (LUC) reporter gene driven by the minimal TATA box of the 35S promoter plus five GAL4 binding elements was used for self-activation test. The 2 kb fragments of upstream sequence from start codon of the candidate genes were amplified and fused with LUC protein to generate reporter plasmids for targets transactivation analysis. A *pTRL* plasmid containing *Renilla LUC* gene driven by the CaMV (Cauliflower mosaic virus) 35S promoter, was used as an internal control. The *pTRL*, effector and reporter were simultaneously transformed into the rice protoplast system, then kept in dark for 16 h. The LUC activity was measured as described previously (Ohta et al., 2000).

### Yeast one-hybrid assay

The *OsSND2*, *OsMYB61L* and *OsMYB86L* encoding sequence was amplified and inserted into the unique EcoRI and XhoI sites of the *pB42AD* vector (Takara) to construct effector. For the reporter vectors, the 2 kb DNA fragments corresponding to the promoter of candidate genes were amplified and cloned into the *pLacZi2μ* vector to drive *lacZ* reporter gene expression. The effectors and reporters were simultaneously transformed into the yeast strain EGY48. The transformants were grown on synthetic dropout plates without tryptophan and uracil containing 5-bromo-4-chloro-3-indolyl-β-D-galactopyranoside for colony coloration. The empty *pB42AD* and *pLacZi* were used as negative control.

### Cell wall composition analysis

The second internodes of wild type and transgenic plants at mature stage were ground into powder under liquid nitrogen and prepared alcohol-insoluble residues (AIRs). De-starched AIRs and trifluoroacetic acid (TFA) treatment were performed as previously described (Li et al., 2009). For the cellulose measurement, the remains after TFA treatment were hydrolyzed in Updegraff reagent (acetic acid: nitric acid: water, 8:1:2 *v*/v). The cooled pellets were washed and hydrolyzed with 72% sulfuric acid. The cellulose content was measured by the anthrone assay (Updegraff, 1969). The monosaccharide composition was determined by gas chromatography-mass spectrometry as described previously (Xiong et al., 2010). The lignin content was measured by the acetyl bromide method as described previously (Huang et al., 2015).

### Microscopy

For the scanning electron microscope (SEM) observation, the second internodes segments were sliced with Gillette razor blades and then fixed in 4% paraformaldehyde. After dehydration through a gradient of ethanol and critical point drying, the samples were sprayed with gold particles and observed with a scanning electron microscope (SEM) (S-3000 N; Hitachi, Tokyo, Japan).

### Accession numbers

Sequence data used in this manuscript can be found in the rice genome annotation database (http://rice.plantbiology.msu.edu) and in the *Arabidopsis* information resource (TAIR, http://www.arabidopsis.org) under the following accession numbers: *OsMYB61* (Os01g18240), *OsSND2* (Os05g48850), *OsCESA4* (Os01g54620), *OsCESA7* (Os10g32980), *OsCESA9* (Os09g25490), *OsCESA11* (Os06g39970), *OsMYB86L* (Os08g36460), *OsMYB61L* (Os05g04820), *AtSND2* (At4g28500).

## Results

### Identification of the interaction between OsSND2 and *OsMYB61* promoter

**Fig. 1:**
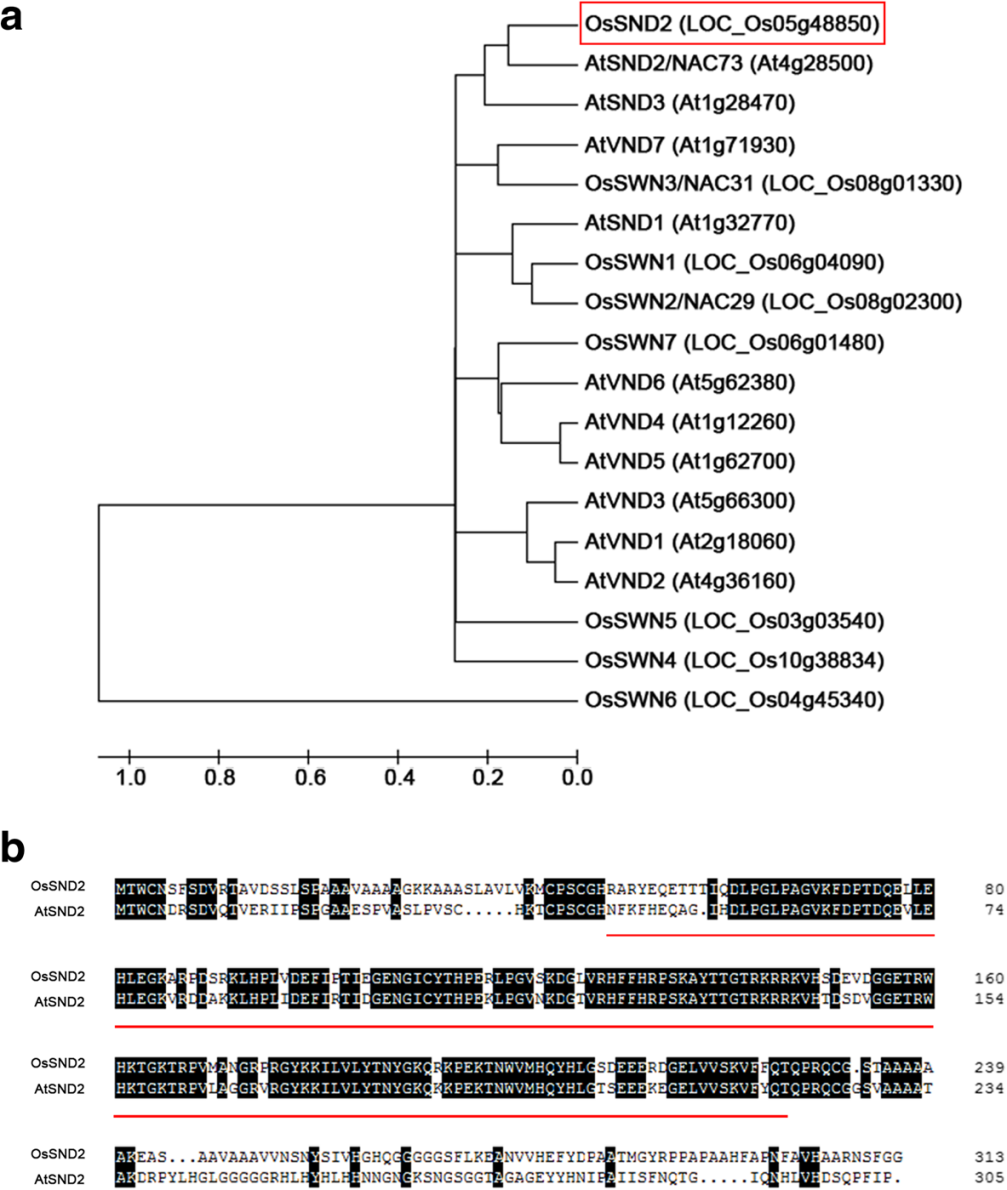
OsSND2 is a NAC family transcription factor and has a very high homology to AtSND2. **a** Phylogenetic analysis of the secondary wall NACs in Rice and Arabidopsis. The red rectangle indicate the OsSND2. An unrooted phylogenetic tree was generated with the full-length amino acid sequences. **b** Protein sequences alignment of OsSND2 and AtSND2. Black shadings indicate identical amino acids. The red underline indicate NAC domain

To understand the hierarchical regulatory mechanism controlling SCWs biosynthesis in rice, we conducted the yeast one-hybrid screening using five different length sequences of *OsMYB61* promoter (Additional file 1: Figure S1a) fused to *HIS3* reporter as baits (Additional file 1: Figure S1b) to search for novel transcription factors involved in the regulation of *OsMYB61* expression. The cDNA library from the second internodes harvested during the heading stage of rice fused to yeast GAL4 activation domain (AD) was used as a prey. To test the bait construct self-activation, promoter bait strains were grown on the SD/−Trp -His media in the presence of 0, 10, 30 and 50 mM of 3-AT, a competitive inhibitor of HIS3 protein. As a result, only the yeast strain with OsMYB61-P5 bait construct was completely suppressed in the presence of 30 mM 3-AT (Additional file 1: Figure S1c). Yeast strains harbouring the other four constructs were not suppressed even with 50 mM of 3-AT (Additional file 1: Figure S1c). Hence, we chose the construct OsMYB61-P5 to perform screening experiment with 30 mM 3-AT. Through the screening of 3.2 × 10^5^ cDNA clones, one positive clone was obtained (clone 13). We isolated the yeast plasmid and subjected it to sequencing and BLAST search against NCBI database (http://blast.ncbi.nlm.nih.gov/Blast.cgi). The BLAST search found a the rice full-length cDNA, NM_001062858. Further sequence analysis and annotation of this clone using RGAP database (http://rice.plantbiology.msu.edu/) showed that this gene is on the locus LOC_Os05g48850, which has three exons and two introns. LOC_Os05g48850 encodes for a NAC family transcription factor with a length of 314 amino acids and a molecular mass of approximately 35 kD. Phylogenetic analysis showed that LOC_Os05g48850 is closely related to NAC family transcription factors in *Arabidopsis* At4g28500 (AtSND2) (Fig. 1a). Protein sequence alignment revealed that they are highly conserved in the predicted NAC DNA-binding domains (Fig. 1b). Therefore, we designated LOC_Os05g48850 as *OsSND2* (*Oryza sativa SND2*).

**Fig. 2:**
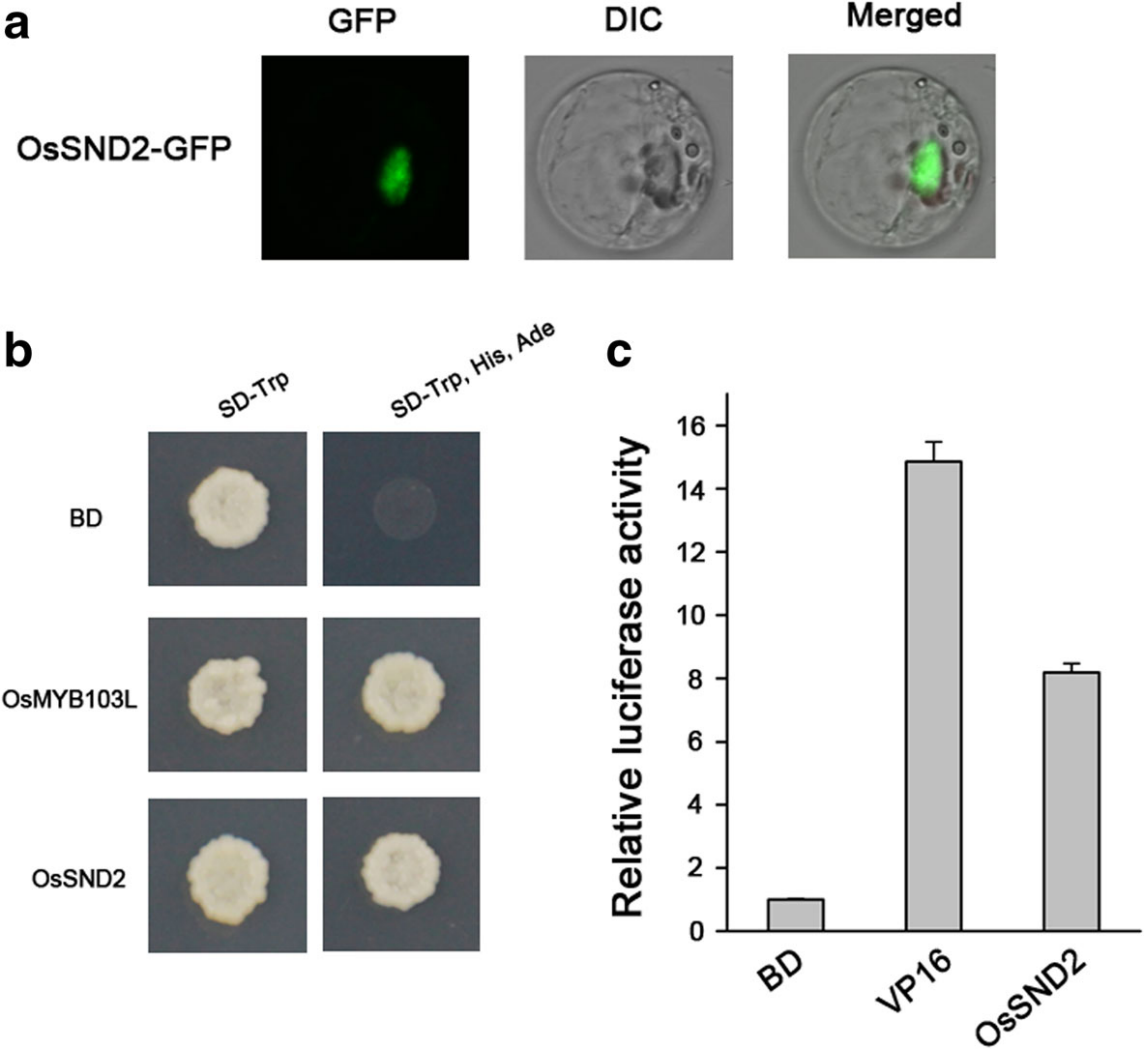
Subcellular localization and transactivation analysis of OsSND2. **a** OsSND2 is a Nuclear-localized protein. A rice protoplast cell expressing OsSND2-GFP, indicating that OsSND2 is a Nuclear-localized protein. **b** Transactivation analysis of OsSND2 fused with the GAL4 DNA binding domain in yeast. Transformants harbouring *pGBKT7-SND2*, the positive control *pGBKT7-MYB103L* and the negative control *pGBKT7* were streaked onto SD-Trp or SD-Trp, His, Ade medium to determine growth. **c** Transactivation analysis of OsSND2 as revealed by relative LUC activity in rice protoplasts

One of the significant features of transcription factors is nuclear localization. To determine the subcellular localization of OsSND2, the construct of OsSND2 with C-terminus green fluorescent protein (GFP) tag was cloned into a *35S::OsSND2-GFP* vector. Using confocal laser scanning microscopy, we confirmed that the OsSND2-GFP fusion protein was located predominantly in the nucleus (Fig. 2a).

To investigate whether OsSND2 has a potential transcriptional activity, we used the yeast assay system to investigate OsSND2. The growth of transformants carrying *pGBKT7-OsSND2* on selective medium (SD/−Trp) and (SD/−Trp –His -Ade) indicated the OsSND2 protein has transcriptional activity, the *pGBKT7*-OsMYB103L and empty *pGBKT7* were used as positive and negative control, respectively (Fig. 2b). We also used a dual-luciferase reporter (DLR) assay system in the rice protoplast to test the transcriptional activation of OsSND2. In comparison with the GAL4-BD negative control, OsSND2 can activate the *LUC* gene, similarly to the activation by VP16 as positive control (Fig. 2c). These results indicate that OsSND2 protein exhibits transcriptional activity (Fig. 2b and c).

### OsSND2 can directly bind to the promoter of *OsMYB61*

**Fig. 3:**
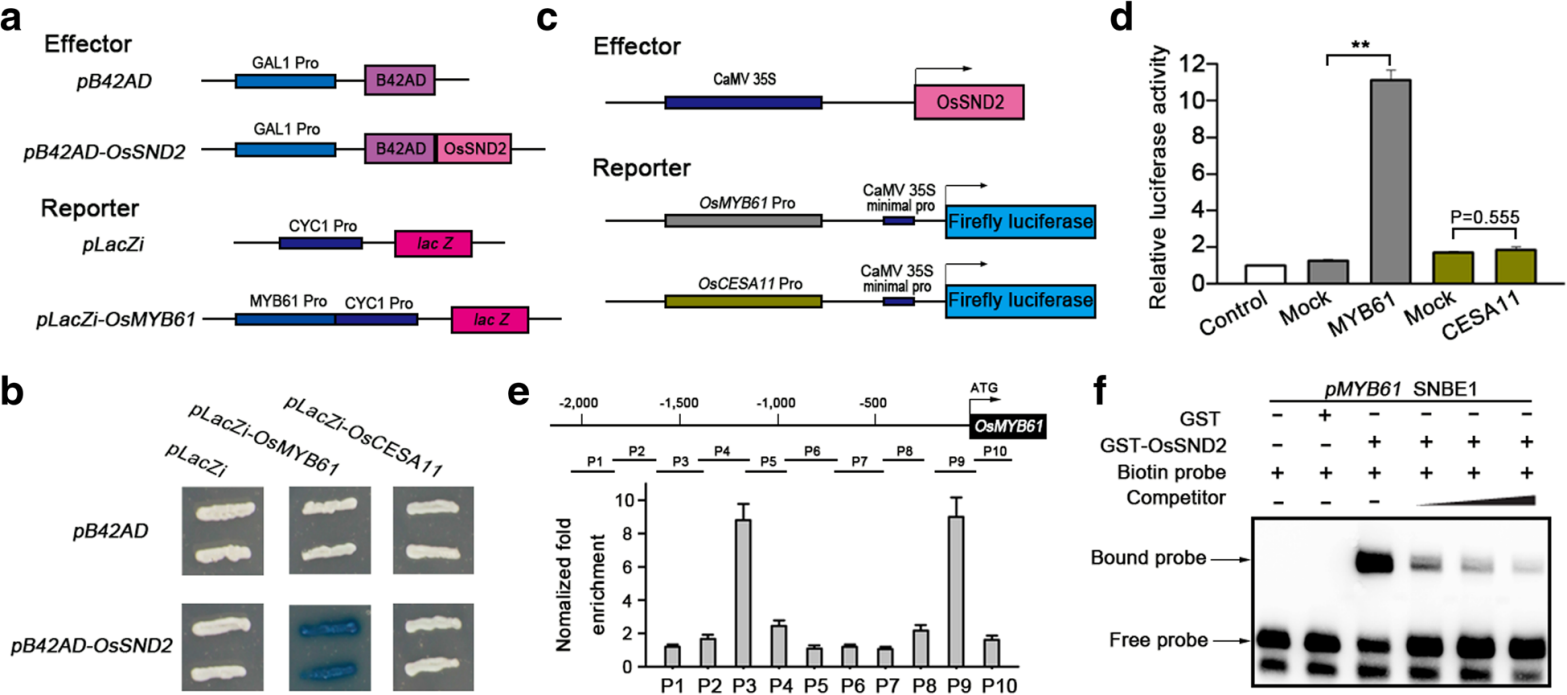
OsSND2 directly regulate *OsMYB61* expression. **a** Diagram of the effector construct and the reporter constructs used in **b**. **b** Yeast one-hybrid assay showing the activity of LacZ reporters driven by *OsMYB61* and *OsCESA11* promoters and activated by activation domain (AD) fusion effectors. The empty *pB42AD* and *pLacZi* were used as negative control. **c** Diagram of the effector construct and the reporter constructs used in **d**. **d** OsSND2 activates transcription of the *OsMYB61* gene promoter. Relative luciferase activity was monitored in rice protoplasts cotransfected with different effector and reporter constructs. Mock, cotransfected with reporter construct and an empty effector construct; control, cotransfected with effector construct and an empty reporter construct (set to 1). Error bars, SE of three biological replicates. Student’s *t*-tests were used to generate the *P* values. The asterisks (**) indicate *p* < 0.01. **e** ChIP assays. The diagram depicts the regions used for ChIP-qPCR analysis of extracts from the second internodes of over-expression plants carrying the *p35S::Myc-OsSND2* construct. ChIP-qPCR results were quantified by normalization of the Myc immunoprecipitation signal by the corresponding input signal. Error bars, SE of three biological replicates. **f** EMSA analysis. Competition for OsSND2 binding was performed with unlabelled P9 fragments containing SNBE1 site at 50×, 100× and 200× the amount of labeled probe

To confirm the interaction between OsSND2 and *OsMYB61* promoter, we used the yeast one-hybrid system with *LacZ* reporter gene (Fig. 3a). The yeast one-hybrid assay revealed the predominant activation of *LacZ* reporter gene expression by OsSND2 under the control of OsMYB61 promoter. On the contrary, pB42AD without OsSND2 failed to activate LacZ expression (Fig. 3b).

We further performed the dual-luciferase reporter (DLR) assay system in rice protoplasts to explore the effect of OsSND2 on the transcriptional regulation of *OsMYB61* expression using a reporter construct carrying the firefly luciferase (LUC) driven by the 2 kb fragment of *OsMYB61* promoter. DLR assay revealed an 11-fold increase in the transcriptional activation in the protoplasts co-expressing an effector carrying OsSND2 (Fig. 3c) and a reporter containing *OsMYB61* promoter to drive luciferase as compared with the negative control (Fig. 3d). This result suggests that OsSND2 functions as a transcriptional activator to directly regulate OsMYB61 expression.

Secondary wall-related NAC proteins regulate target genes expression through binding to the SNBE element, (T/A)NN(C/T)(T/C/G)TNNNNNNNA(A/C)GN(A/C/T)(A/T) (Zhong et al., 2010). To determine whether the interaction between OsSND2 and OsMYB61 promoter occurs through binding to SNBE site, we performed sequence searching within the promoter of OsMYB61 and found it contains two SNBE sites (SNBE1 and SNBE2) (Additional file 2: Figure S2a). We further conducted chromatin immunoprecipitation (ChIP) assay in wild-type and *p35S::Myc-OsSND2* overexpression transgenic rice plants. The results showed that the two fragments (P3 and P9) containing the SNBE sites were significantly enriched in *Myc-OsSND2* overexpression plants (Fig. 3e). We used an electrophoretic mobility shift assay (EMSA) to examine whether OsSND2 bind to P9 and P3 fragments containing the SNBE1 and SNBE2, respectively. P9 and P3 were bound by the recombinant OsSND2 protein fused to glutathione *S*-transferase (GST-OsSND2), which resulted in a mobility shift (Fig. 3f and Additional file 2: Figure S2b). GST alone, as a negative control, failed to induce the mobility shift (Fig. 3f and Additional file 2: Figure S2b). The binding ability to two fragments was gradually decreased in the presence of increasing amounts of unlabeled probes (Fig. 3f and Additional file 2: Figure S2b), thereby confirming the binding specificity.

Taken together, the above results demonstrate the function of OsSND2 as a transcription activator through its direct binding to SNBE sites in the promoter of *OsMYB61* in vitro and in vivo.

### *OsSND2* was mainly expressed in internodes and panicles

**Fig. 4:**
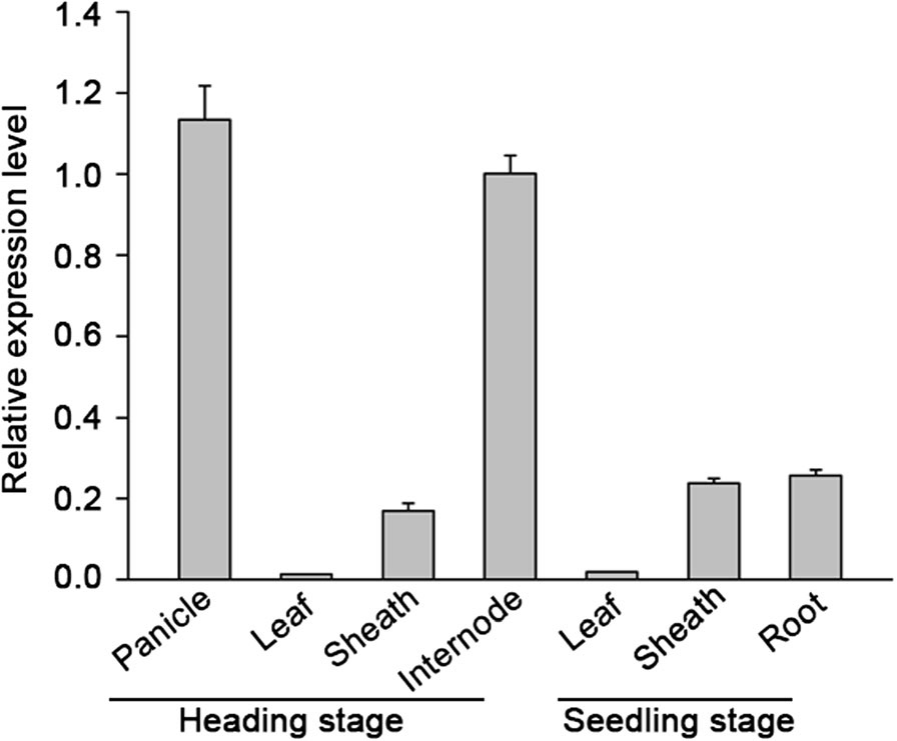
Expression pattern of *OsSND2*. qRT-PCR analysis of *OsSND2* expression in various rice organs and different developmental stage, the heading stage and seedling stage indicate the tenth day after flowering and the two weeks old seedlings, respectively. The *Actin1* gene was used as an internal control. Error bars, SD of three biological replicates

To investigate whether the expression of OsSND2 is associated with SCWs biosynthesis, the expression pattern of *OsSND2* was examined by quantitative real-time PCR (qRT-PCR) using RNAs isolated from various organs of WYJ7 plants. *OsSND2* expression was detected in all organs, with relatively higher levels observed in internodes and panicles. The expression level of *OsSND2* was relatively low in leaves, sheaths, and roots during the heading and seedling stages (Fig. 4). We examined the expression pattern of *OsMYB61* and found it to be consistent with *OsSND2* expression (Additional file 3: Figure S3).

### Mutation of OsSND2 decreased cellulose content and down-regulated SCWs gene expression

**Fig. 5:**
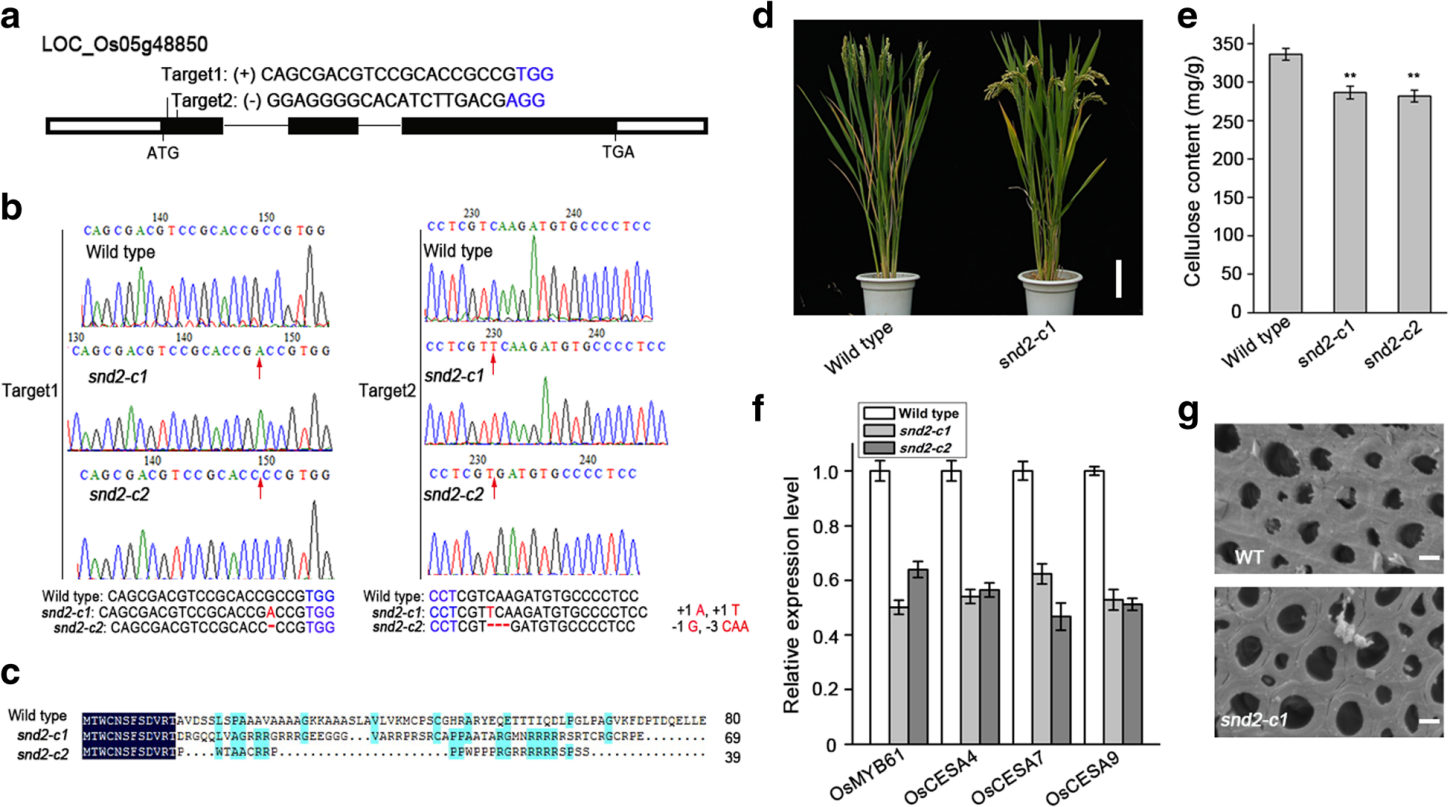
Generation and analysis of *snd2* mutants. **a** Schematic diagram of *OsSND2* gene structure and two CRISPR/Cas9 target sites. UTRs, exons, and introns are indicated by blank rectangles, black rectangles, and black lines, respectively. **b** DNA sequence alignments for the two homozygous *snd2* mutants identified in the T1 generation, together with a wild-type (WT) control. The numbers on the right side are the sizes of the indels, with “−” and “+” showing deletion and insertion of nucleotides involved, respectively. **c** Deduced OsSND2 amino acid sequence alignments for the two homozygous mutants and WT. **d** Three-month-old plant of wild type (WT) and *snd2-c1* mutant. Bar = 10 cm. **e** Measurement of cellulose content in WT and *snd2* mutants. **f** Relative expression of *OsMYB61* and SCWs-related *CESA* genes in WT and *snd2* mutants. The *Actin1* gene was used as internal control. Error bars, SD of three biological replicates. **g** Observation of sclerenchyma cell walls in the internodes from the three-month-old wild-type and *snd2* mutant plants via transmission electron microscope. Bar = 2 μm

To investigate the biological function of OsSND2, we generated *OsSND2* mutants using CRISPR/Cas9 system. We designed two sequence-specific single guide RNA (sgRNA) target sites, Target1 and Target2, which were 76-bp apart in the first exon of *OsSND2* (Fig. 5a). Two transgene-free homozygous knockout lines with different genotypes, *snd2-c1* and *snd2-c2* were obtained (Fig. 5b). Protein sequence alignments of the two homozygous mutants and the wild type protein revealed that *snd2-c1* and *snd2-c2* showed coding frame shifts and premature translational stops (Fig. 5c).

No obvious morphological changes, except for a little early flowering in *ossnd2* mutant (Fig. 5d). However, a significant decrease in the cellulose content was detected in *snd2-c1* and *snd2-c2* mutants (Fig. 5e). No significant alteration in the contents of xylose and lignin were detected (Additional file 4: Table S1) We determined the expression levels of *OsMYB61* and secondary wall *CESA* genes in two mutants and found that the expression levels of *OsMYB61* and *CESA* genes were down-regulated (Fig. 5f). We further analyzed the wall thickness of sclerenchyma cells in the internodes of wild-type and *snd2* mutant plants by SEM and found that *snd2* mutant plants showed obviously thinner walls than the wild-type plants in sclerenchyma cells (Fig. 5g).

### Overexpression of *OsSND2* increased cellulose content and up-regulated SCWs gene expression

**Fig. 6:**
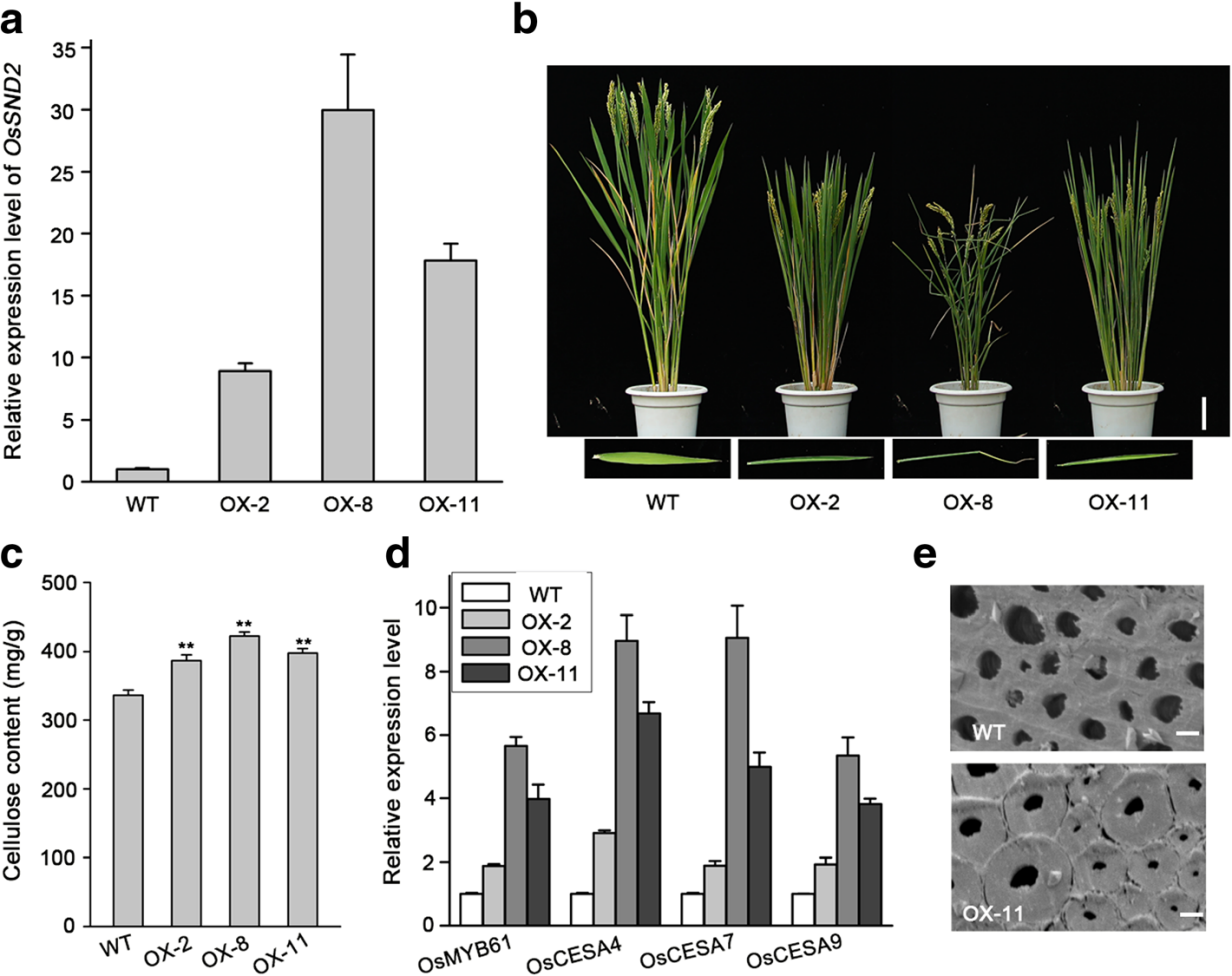
Over-expression of *OsSND2* results leaf rolling phenotype and increases cellulose content. **a** Three-month-old plant of wild type (WT) and over-expression (OX) transgenic lines. Bar = 10 cm (top panel). The OX lines showed upward rolled leaves compare to WT (bottom panel). **b** *OsSND2* expression in over-expression (OX) transgenic plants as determined by qRT-PCR. The *Actin1* gene was used as internal control. Error bars, SD of three biological replicates. **c** Measurement of cellulose content in WT and OX lines. Cellulose content (milligrams per gram of total cell wall residues) of the second internodes from WT and OX plants. Error bars, SD of three biological replicates. The asterisks (**) indicate a significant difference between transgenic plants and WT controls at *P* < 0.01, by Student’s *t*-test. **d** Relative expression of *OsMYB61* and SCWs-related *CESA* genes in WT and OX lines was determined by qRT-PCR analysis. The *Actin1* gene was used as an internal control. Error bars, SD of three biological replicates. **e** Observation of sclerenchyma cell walls in the internodes from the three-month-old wild-type and SND2- OX plants via transmission electron microscope. Bar = 2 μm

To further elucidate the biological function of OsSND2, we generated *OsSND2* overexpression (OX) transgenic plants. Seventeen OX transgenic lines were obtained. We used qRT-PCR analysis to examine the expression level of *OsSND2* in these transgenic plants. Transgenic lines with significant alterations in the expression level of *OsSND2* were selected for further study (Fig. 6a).

The overexpression of OsSND2 resulted in the phenotypic characteristics such as semi-dwarf plant height and significant leaf rolling. The degree of leaf rolling increased with an increase in the expression level of *OsSND2* (Fig. 6b). We measured the cellulose content in *OsSND2*-OX plants and found that *OsSND2*-OX plants have significantly increased cellulose content (Fig. 6c), but showed no significant alterations in the contents of xylose and lignin (Additional file 4: Table S1). We further examined the expression levels of *OsMYB61* and secondary wall *CESA* genes using qRT-PCR. Consistent with the increased cellulose content, *OsMYB61* and *CESA* genes expression level were higher in *OsSND2*-OX plants (Fig. 6d). The results of anatomical analysis revealed the obvious thickness in the sclerenchyma cell wall of OsSND2-OX plants as compared to wild-type plants (Fig. 6e). Collectively, these results suggest that OsSND2 may be involved in the regulation of the biosynthetic pathways involved in SCW cellulose synthesis and affect the thickness of sclerenchyma cell wall.

### OsSND2 directly regulates the expression of other R2R3-MYB family TFs

**Fig. 7:**
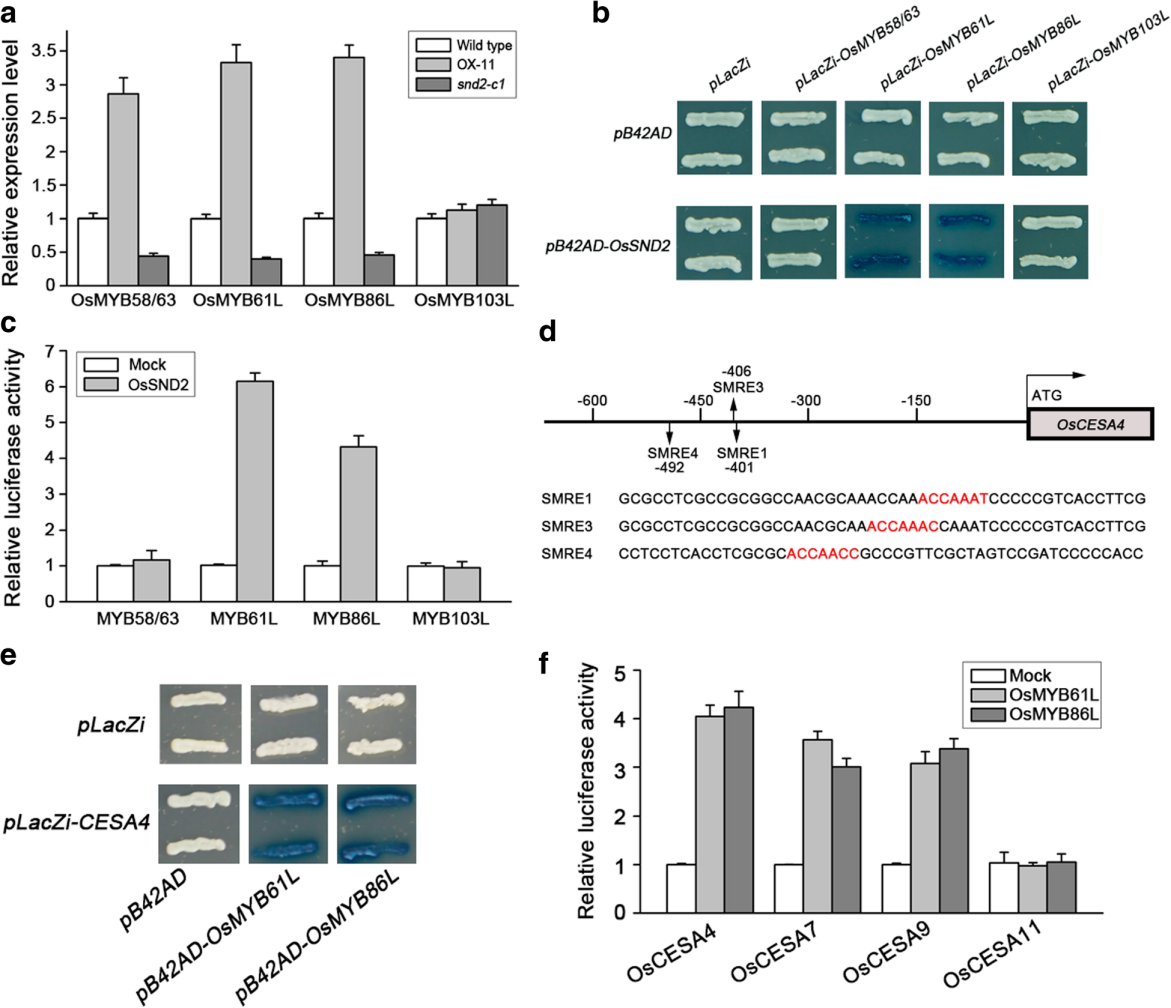
OsSND2 and *R2R3-MYB*s regulates its target genes expression. **a** Relative expression of *R2R3-MYB*s genes in WT, OsSND2-OX and *snd2-c1* mutant lines. The *Actin1* gene was used as internal control. Error bars, SD of three biological replicates. **b** OsSND2 directly binds to *OsMYB61L* and *OsMYB86L* promoters. Yeast one-hybrid assay showing the activity of LacZ reporters driven by *OsMYB58.63*, *OsMYB61L*, *OsMYB86L, OsMYB103L* promoters and activated by activation domain (AD) fusion effectors. The empty *pB42AD* and *pLacZi* were used as negative control. **c** OsSND2 activates transcription of the *OsMYB61L* and *OsMYB86L* gene promoter. Luciferase activities in rice protoplasts contransfected with the effectors and reporters. The transactivation activity was monitored by assaying the luciferase activities, with the activity in protoplasts transfected with an empty effector construct defined as 1. Error bars, SD of three biological replicates. **d** Diagram of *OsCESA4* showing three SMRE elements within the 600 bp *OsCESA4* promoter region. Black arrows indicate the SMRE elements. The DNA sequences containing a core motif of SMREs (the red bases). The DNA sequence containing these three SMREs was subjected to the EMSA assay. **e** Yeast one-hybrid assay showing the activity of LacZ reporters driven by *OsCESA4* promoter containing three SMRE sites and activated by activation domain (AD) fusion effectors. The empty *pB42AD* and *pLacZi* were used as negative control. **f** OsMYB61L and OsMYB86L activate transcription of the *OsCESA4*, *OsCESA7* and *OsCESA9* genes promoter. Luciferase activities in rice protoplasts contransfected with the effectors and reporters. The transactivation activity was monitored by assaying the luciferase activities, with the activity in protoplasts transfected with an empty effector construct defined as 1. Error bars, SD of three biological replicates

Secondary wall-related NAC proteins can activate lots of R2R3-type *MYB* family TFs expression to start the entire transcription regulation network controlling SCWs biosynthesis in *Arabidopsis* (Zhong et al., 2008). To investigate whether OsSND2 regulates other SCWs-related R2R3-MYB family TFs expression in rice, we performed co-expression analysis of OsSND2 with SCWs-related *CESAs* and R2R3-type *MYB*s. We identified several MYB candidates that may be involved in SCWs biosynthesis (Table 1). We further examined the expression levels of these R2R3-type MYBs in *snd2-c1* mutants and *OsSND2* overexpression transgenic plants. qRT-PCR assay showed that *OsMYB86L* (LOC_Os08g36460), *OsMYB61L* (LOC_Os05g04820), and *OsMYB58/63* (LOC_Os04g50770) were down-regulated in *snd2-c1* mutants and up-regulated in the transgenic *OsSND2*-OX plants (Fig. 7a). The expression of *OsMYB103L* (LOC_Os08g05520) had no obvious difference in these transgenic lines (Fig. 7a).

**Table 1:** List of the *R2R3-MYB* TFs and secondary wall *CESA* genes coexpressed with *OsSND2* in Rice

Gene type	Gene	Gene ID	PCC
Cellulose biosynthesis genes	*OsCESA4*	LOC_Os01g54620	0.8475
	*OsCESA7*	LOC_Os10g32980	0.8292
	*OsCESA9*	LOC_Os09g25490	0.8404
R2R3-type MYBs	*OsMYB103L*	LOC_Os08g05520	0.7664
	*OsMYB86L*	LOC_Os08g36460	0.7624
	*OsMYB61*	LOC_Os01g18240	0.7503
	*OsMYB61L*	LOC_Os05g04820	0.6899
	*OsMYB58*/63	LOC_Os04g50770	0.6770

Co-expression analysis of OsSND2 with *R2R3-MYB* TFs and secondary wall *CESA* genes was performed using the expressing database at http://www.ricearray.org/coexpression/coexpression.shtml. 1, The Pearson correlation coefficient (PCC). The PCC of coexpressed R2R3-MYBs and SCWs-related CESA genes was set above 0.65 and 0.75, respectively

To further investigate if OsSND2 can directly regulate the expression of these MYBs, transcriptional activation assays were performed in yeast and rice protoplasts. *OsMYB86L* and *OsMYB61L,* but not *OsMYB58/63* were directly regulated by OsSND2 (Fig. 7b and c). In addition, we investigated the distribution of SNBE sites and found two and three SNBE sites in the promoter region (1.5-kb 5′-upstream sequence of the start codon) of *OsMYB86L* and *OsMYB61L*, respectively. Hence, OsSND2 directly regulates the expression of *OsMYB86L* and *OsMYB61L,* probably through its binding to SNBE sites in the promoter regions of *OsMYB86L* and *OsMYB61L*.

### OsMYB86L and OsMYB61L directly activate the transcription of *CESAs*

OsCESA4, OsCESA7 and OsCESA9 are essential for SCWs cellulose biosynthesis in rice. To investigate whether OsMYB86L and OsMYB61L are involved in SCWs biosynthesis, we performed yeast one-hybrid using pB42AD-(OsMYB86L and OsMYB61L) fusion proteins and *OsCESA4* promoter region (− 600 to − 1 bp upstream of the start codon), which contains three SCWs MYB-responsive elements (SMRE) (Fig. 7d). These results provided evidence that OsMYB61L and OsMYB86L bind to the promoter region of *OsCESA4* (Fig. 7e).

We performed the transcriptional activation assay in rice protoplasts to investigate whether OsMYB86L and OsMYB61L can activate the transcription of *OsCESA4*, *OsCESA7* and *OsCESA9*. The assay results show that the luciferase activity was significantly higher for protoplasts co-expressing the reporter carrying three *CESA* gene promoters driving luciferase and the effector containing OsMYB86L or OsMYB61L than the negative control (Fig. 7f). Thus, OsMYB86L and OsMYB61L directly activate the transcription of *OsCESA4*, *OsCESA7*, and *OsCESA9*.

## Discussion

Secondary cell walls play a critical role in plant growth and development, and they also contain high amounts of lignocellulose, a key feedstock for the production of bio-energy and bio-based products. In rice, OsMYB61 is a key regulator that binds to the promoter of *CESA* genes and regulates their expression (Huang et al., 2015). Yeast one-hybrid screening is a powerful tool for the identification and isolation more transcription factors using promoter segments or regulatory elements of targets as baits. In this study, we isolated a NAC transcription factor from the yeast one-hybrid screening using OsMYB61 promoter region containing a SNBE site as bait (Additional file 1: Figure S1). We named it as the OsSND2 based on its closed relationship with AtSND2. We confirmed the direct binding of OsSND2 on *OsMYB61* promoter in vitro and in vivo (Fig. 3 and Additional file 2: Figure S2). We also further investigated OsSND2 protein function and downstream genes.

### OsSND2 directly activate *OsMYB61* expression

We have demonstrated that OsSND2 can directly bind to the promoter of *OsMYB61* (Fig. 3b and d). In *Arabidopsis*, secondary wall NAC family proteins (SWNs) activate their direct target genes through binding to the SNBE sites, and the binding affinities vary with different SWNs and SNBE sequences (McCarthy et al., 2014; Zhong et al., 2010). We have found two SNBE sites in *OsMYB61* promoter region. EMSA and ChIP assay showed that OsSND2 binds to the two SNBE sites (Fig. 3e, f and Additional file 2: Figure S2). In previous study, NAC29 and NAC31 were shown to bind only to the SNBE site farther from the start codon (Huang et al., 2015).

The NAC family transcription factors are highly conserved at the N-terminal NAC binding domain and have a highly variable C-terminal domain, which may function as a transcriptional activator or repressor (Olsen et al., 2005). A large number of SWNs function as a transcriptional activator to regulate downstream genes expression in *Arabidopsis*, such as SND1 and its close homologs (Zhong et al., 2008). Transactivation analysis indicated that OsSND2 exhibits transcriptional activity (Fig. 2b and c) and functions as a transcriptional activator to initiate the transcription of *OsMYB61* (Fig. 3d). The expression of *OsMYB61* was up-regulated in *OsSND2*-OX transgenic lines (Fig. 6d) and down-regulated in *snd2* mutants (Fig. 5f). We also detected similar expression patterns for *OsSND2* and *OsMYB61* (Fig. 4 and Additional file 3: Figure S3). Previous study shows that the transcription of *OsMYB61* is mainly mediated by NAC29 and NAC31 (Huang et al., 2015). Therefore, we reported OsSND2 as a new transcriptional activator to directly regulate *OsMYB61* expression.

### OsSND2 regulate secondary wall cellulose biosynthesis

In rice, the genome was predicted to contain 151 *NAC* genes (Nuruzzaman et al., 2010). The NAC family transcription factors play important roles in plant growth and development (Olsen et al., 2005), especially in response to different abiotic stresses (Fujita et al., 2004; Hegedus et al., 2003; Tran et al., 2004) and SCWs formations (Zhong and Ye, 2015), NAC proteins may have contributed to the evolution of both water-conducting and supporting cells during the adaptation of plants to land (Xu et al., 2014). In *Arabidopsis*, SWNs can originate entire regulation network controlling SCWs biosynthesis, SND1 and its homologs act directly upstream of MYB46 and MYB83, which have been reported to bind the promoter of SCWs *CESA*s to regulate SCWs cellulose biosynthesis (Wang and Dixon, 2012). OsCESA4, OsCESA7 and OsCESA9 are responsible for secondary wall cellulose biosynthesis in rice, mutation or down-regulation expression of these *CESA* genes results in brittle culm phenotype and reduction of the cellulose content (Kotake et al., 2011; Tanaka, 2003; Zhang et al., 2009). OsMYB61 has been reported to directly bind to the promoters of *CESA* genes to regulate their expression and cellulose biosynthesis (Huang et al., 2015). *NAC29*-OX, *NAC31*-OX and *OsMYB61*-OX transgenic plants have thick internodes, upward curved leaves, and significantly increased cellulose content (Huang et al., 2015). Our findings revealed the up-regulated and down-regulated expression of *OsMYB61* in *OsSND2*-OX lines (Fig. 6d) and *snd2* mutants (Fig. 5f), respectively. Consistent with the expression level of *OsMYB61*, up-regulation of *CESA* genes expression (Fig. 6d) and increased cellulose content were observed in *OsSND2*-OX lines (Fig. 6c). On the other hand, down-regulated *CESA* genes expression (Fig. 5f) and decreased cellulose content were found in *snd2* mutants (Fig. 5e). Unlike OsMYB103L, whose mutation lead to the reduction in the cellulose content and a brittle culm phenotype (Ye et al., 2015), *snd2* mutants showed normal culm and no obvious change in plant morphology except for lower cellulose content and thinner sclerenchyma cell wall(Fig. 5b and g). These observations suggest that the function of OsSND2 may be redundant to its close homolog in rice. The contents of xylose and lignin were almost unchanged in *snd2* mutants and SND2-OX plants (Additional file 4: Table S1). Thus, OsSND2 functions as a regulator to control SCWs cellulose biosynthesis.

### Hierarchical transcriptional network regulating the SCWs biosynthetic program is present in rice

In *Arabidopsis*, the detailed transcriptional network regulating the SCWs biosynthesis has been revealed (Zhong and Ye, 2015). In this transcriptional network, the secondary wall NAC families (SWNs) function as the top-layer master switches to regulate a battery of downstream transcription factors, including *SND2*, *SND3*, *MYB20*, *MYB42*, *MYB46*, *MYB83* and *MYB103* to start the entire SCWs biosynthetic program (Zhong et al., 2008). MYB46 and MYB83 function as the regulators of the secondary-layer and regulate the expression of other MYBs and biosynthetic genes for cellulose, xylan and lignin (Zhong and Ye, 2012). A few studies have investigated the hierarchical transcriptional network regulating SCWs formation in rice. We demonstrated that OsSND2 functions as a regulator to control the expression of MYBs (Fig. 7a), which can further activate the SCWs *CESA* genes expression. Furthermore, we proved that AtSND2 (At4g28500) can bind to the promoter of *AtMYB61* (At1g09540) in yeast one-hybrid assay (Additional file 5: Figure S4). This result suggests the conservation of the regulatory mechanism in dicot and monocot plants. In *Arabidopsis*, AtSND2 acts downstream of AtSND1 and its close homologs (Zhong et al., 2008). As OsSWN1 and OsSWN2/OsNAC29 are close homologs of AtSND1 in rice (Fig. 1a), they may function as regulators to activate OsSND2 expression and initiate the entire SCWs biosynthetic program. OsMYB58/63, OsMYB61L and OsMYB86L act downstream of OsSND2 (Fig. 7). We have demonstrated that OsMYB61L and OsMYB86L directly activate the transcription of SCWs-related *CESA* genes (Fig. 7f). OsMYB58/63 was shown to directly up-regulate the expression of *OsCESA7* (Noda et al., 2015). Therefore, OsSND2 may act as the secondary-layer master switch involved in the controlling of SCWs biosynthesis, thus, a hierarchical transcriptional network similar to that of *Arabidopsis* also exists in rice (Additional file 6: Figure S5). These results are consistent with the previous study for survey of involved in rice SCWs formation through a co-expression network (Hirano et al., 2013).

### *OsSND2* has a potential value in rice straw management

As one of the most important staple food crops, rice produces huge amount of agronomic biomass residues. The handling of biomass is a challenge for breeders, as rice straws decomposition take a long time. Farmers prefer straw burning, which is economic and convenient, but may causes environmental problems. The major reason underlying the difficult treatment procedure is the high cellulose content of the cell wall of straws (Tian et al., 1992). The *brittle culm* (*bc*) rice mutants are the ideal breeds for straws treatment owing to lower cellulose content and finer breakage at harvest (Cabiles et al., 2008; Johnson et al., 2006). However, not all *bc* mutants can be used for breeding because of their concomitant phenotypes, such as dwarfism, low fertility and withering of leaf apex (Zhang et al., 2009; Zhang et al., 2010; Zhou et al., 2009). Mutations in OsMYB103L, a TF regulating SCWs-related genes expression, lead to the decreased cellulose content and brittle culm phenotype without morphological abnormalities (Ye et al., 2015). We have demonstrated that OsSND2 can regulate *MYB*s and SCWs *CESA* genes expression (Figs. 5f, 6d and 7a), and that *snd2* mutants have lower cellulose contents (Fig. 5e) and exhibit no change in morphology (Fig. 5d). The *snd2* mutant plants exhibit a little early flowering may be caused by the effects of the flowering genes expression (Fig. 5d), but show normal morphology. Hence, *snd2* mutants have the potential value for rice straws management.

## Conclusion

In this study, *OsSND2* was identified as a positive regulator of cellulose biosynthesis in rice. Increasing the expression level of this gene can improve the SCWs cellulose content, but the content of xylose and lignin were not affected. Therefore, study the function of OsSND2 can provide a strategy for manipulating plant biomass production.

## Additional files

Additional file 1: **Figure S1.** Yeast one-hybrid screening using different fragments of *OsMYB61* promoter as baits. **a**, Diagram of *OsMYB61* with five different fragments using for bait constructs. **b**, Diagram of bait construct in yeast one-hybrid screening. **c**, Self-activation test of five different bait constructs. The transformants harbouring the different bait construct were streaked onto SD-Trp, His media in the presence of 0 mM, 10 mM, 30 mM and 50 mM 3-aminotriazole (3-AT) to determine growth. (TIF 2102 kb)

Additional file 2: **Figure S2.** OsSND2 binds to the SNBE sites in the *OsMYB61*promoter. **a**, Diagram of *OsMYB61* promoter containing two SNBE sites. Dark brown boxes indicate the SNBE elements. The DNA sequences containing the SNBE sites (the red bases) were subjected to the EMSA assay. **b**, EMSA assay showing that the recombinant *OsSND2* protein directly bound to the biotin-labeled sequence containing SNBE2 site. (TIF 1061 kb)

Additional file 3: **Figure S3.** Expression pattern of *OsMYB61*. qRT-PCR analysis of *OsMYB61* expression in various rice organs and different developmental stage, the heading stage and seedling stage indicate the tenth day after flowering and the two weeks old seedlings, respectively. The *Actin1* gene was used as an internal control. Error bars, SD of three biological replicates. (TIF 1176 kb)

Additional file 4: **Table S1.** Composition analysis of sugar and lignin content of wall residues of the internodes from wild type and transgenic rice plants. (DOC 30 kb)

Additional file 5:: **Figure S4.** AtSND2 directly binds to the promoter of *AtMYB61*. Yeast one-hybrid assay showing the activity of LacZ reporters driven by *AtMYB61* promoter (2 kb length sequence from the start codon) and activated by AtSND2 fused with activation domain (AD). The empty *pB42AD* and *pLacZi* were used as negative control. (TIF 331 kb)

Additional file 6:: **Figure S5.** The transcriptional regulatory model of SCW formation in rice. Arrows indicate transcriptional activation, whereas flat-ended arrows indicate transcriptional repression. Solid arrows indicate direct transcriptional activation. Dashed arrows indicate indirect transcriptional activation. (TIF 136 kb)
