# Supplementary figures and images for: OsSND2, a NAC family transcription factor, is involved in secondary cell wall biosynthesis through regulating MYBs expression in rice
Source: Rice (N Y). 2018 May 31;11:36. doi: 10.1186/s12284-018-0228-z (PMC5981155; doi:10.1186/s12284-018-0228-z)

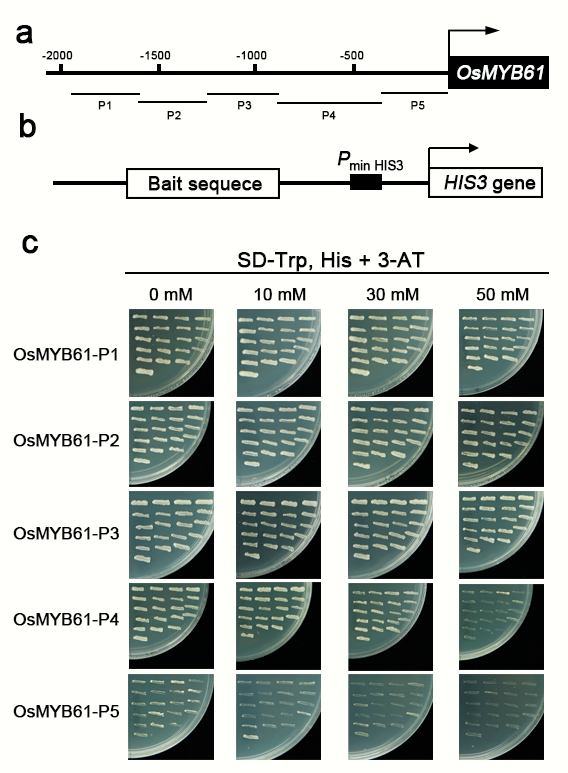

Supplement: Supplementary file 1 — Figure S1. Yeast one-hybrid screening using different fragments of OsMYB61 promoter as baits. a, Diagram of OsMYB61 with five different fragments using for bait constructs. b, Diagram of bait construct in yeast one-hybrid screening. c, Self-activation test of five different bait constructs. The transformants harbouring the different bait construct were streaked onto SD-Trp, His media in the presence of 0 mM, 10 mM, 30 mM and 50 mM 3-aminotriazole (3-AT) to determine growth. (TIF 2102 kb) [file 12284_2018_228_MOESM1_ESM.tif]

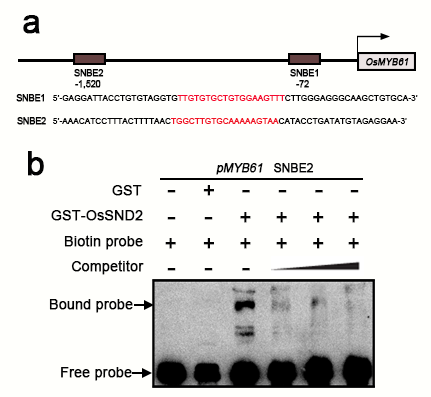

Supplement: Supplementary file 2 — Figure S2. OsSND2 binds to the SNBE sites in the OsMYB61promoter. a, Diagram of OsMYB61 promoter containing two SNBE sites. Dark brown boxes indicate the SNBE elements. The DNA sequences containing the SNBE sites (the red bases) were subjected to the EMSA assay. b, EMSA assay showing that the recombinant OsSND2 protein directly bound to the biotin-labeled sequence containing SNBE2 site. (TIF 1061 kb) [file 12284_2018_228_MOESM2_ESM.tif]

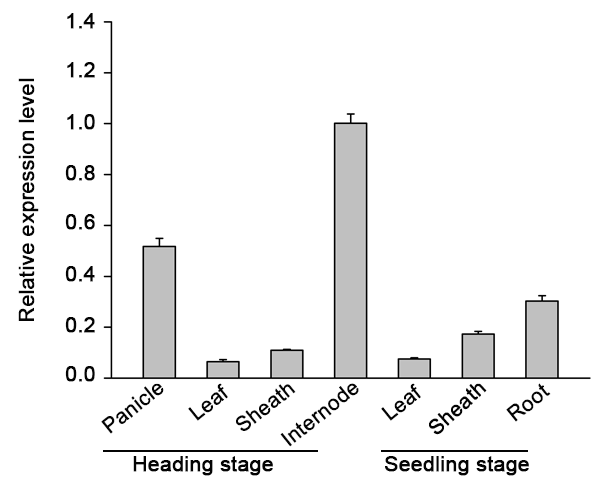

Supplement: Supplementary file 3 — Figure S3. Expression pattern of OsMYB61. qRT-PCR analysis of OsMYB61 expression in various rice organs and different developmental stage, the heading stage and seedling stage indicate the tenth day after flowering and the two weeks old seedlings, respectively. The Actin1 gene was used as an internal control. Error bars, SD of three biological replicates. (TIF 1176 kb) [file 12284_2018_228_MOESM3_ESM.tif]

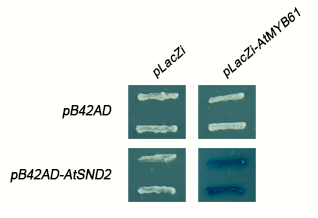

Supplement: Supplementary file 5 — Figure S4. AtSND2 directly binds to the promoter of AtMYB61. Yeast one-hybrid assay showing the activity of LacZ reporters driven by AtMYB61 promoter (2 kb length sequence from the start codon) and activated by AtSND2 fused with activation domain (AD). The empty pB42AD and pLacZi were used as negative control. (TIF 331 kb) [file 12284_2018_228_MOESM5_ESM.tif]

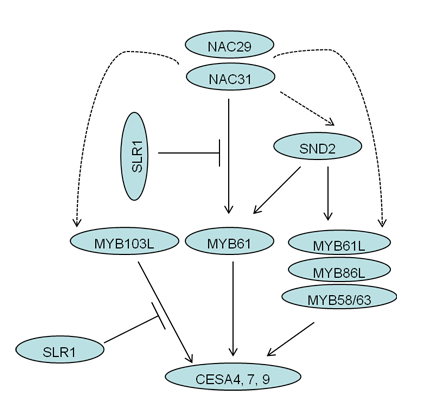

Supplement: Supplementary file 6 — Figure S5. The transcriptional regulatory model of SCW formation in rice. Arrows indicate transcriptional activation, whereas flat-ended arrows indicate transcriptional repression. Solid arrows indicate direct transcriptional activation. Dashed arrows indicate indirect transcriptional activation. (TIF 136 kb) [file 12284_2018_228_MOESM6_ESM.tif]
