# Supplementary material for: OsSND2, a NAC family transcription factor, is involved in secondary cell wall biosynthesis through regulating MYBs expression in rice
Source: Rice (N Y). 2018 May 31;11:36. doi: 10.1186/s12284-018-0228-z (PMC5981155; doi:10.1186/s12284-018-0228-z)
Supplement: Supplementary file 4 — Table S1. Composition analysis of sugar and lignin content of wall residues of the internodes from wild type and transgenic rice plants. (DOC 30 kb) [file 12284_2018_228_MOESM4_ESM.doc]

*Additional file 4: Table S1 Composition analysis of sugar and lignin content of wall residues of the internodes from wild type and transgenic rice plants*

| Sample | Rhamnose | Fucose | Arabinose | Xylose | Mannose | Galactose | Glucose | Lignin |
| --- | --- | --- | --- | --- | --- | --- | --- | --- |
| Wild type | 1.91±0.01 | 0.88±0.01 | 22.42±0.41 | 244.32±4.59 | 1.31±0.01 | 14.35±0.16 | 70.13±0.71 | 128.06±1.33 |
| *snd2-c1* | 2.00±0.11 | 0.85±0.03 | 21.32±0.99 | 245.18±4.73 | 1.34±0.01 | 11.05±0.60 | 70.33±1.87 | 129.11±1.14 |
| SND2-OX2 | 1.96±0.01 | 0.86±0.01 | 22.89±0.31 | 241.45±4.35 | 1.36±0.01 | 15.35±0.26 | 71.25±1.12 | 128.83±1.28 |
| SND2-OX8 | 1.98±0.01 | 0.88±0.01 | 24.49±0.46 | 246.24±4.51 | 1.33±0.01 | 16.54±0.29 | 70.37±1.09 | 129.23±1.65 |
| SND2-OX11 | 1.96±0.03 | 0.86±0.01 | 23.16±0.40 | 245.33±4.18 | 1.35±0.01 | 16.25±0.31 | 70.28±1.16 | 128.92±1.41 |

The results are means ±SE of five independent assays. Each wall component was calculated as mg·g^-1^ of alcohol-insoluble cell-wall residue.
